# PS-341 alleviates chronic low-grade inflammation and improves insulin sensitivity through the inhibition of TM4 (UBAC2) degradation

**DOI:** 10.1186/s12986-021-00579-8

**Published:** 2021-06-01

**Authors:** Lili Chen, Kuanping Ye, Xiaocheng Feng, Lianxi Li, Qin Li, Ying Huang, Xuanchun Wang, Rumei Li, Cheng Hu, Zhen Yang, Bin Lu, Yehong Yang, Jie Wen, Zhaoyun Zhang, Min He, Qinghua Wang, Wenbai Zhou, Yintao Li, Naijia Liu, Jinya Huang, Qiwei Shen, Qiyuan Yao, Renming Hu

**Affiliations:** 1 Department of Endocrinology, Huashan Hospital Fudan University, 12 Middle Wulumuqi Road, Shanghai, 200040 China; 2 Institute of Endocrinology and Diabetology, Fudan University, Shanghai, China; 3 Department of Endocrinology, Sir Run Run Shaw Hospital, School of Medicine, Zhejiang University, Hangzhou, China; 4 Department of Endocrinology, Shanghai Sixth People’s Hospital, Shanghai Jiaotong University, Shanghai, China; 5 Department of Endocrinology, School of Medicine, Shanghai Ninth People’s Hospital, Shanghai Jiaotong University, Shanghai, China; 6 Department of Endocrinology, Shanghai First People’s Hospital, Shanghai Jiaotong University, Shanghai, China; 7 Department of Endocrinology, Xinhua Hospital, School of Medicine, Shanghai Jiaotong University, Shanghai, China; 8 Department of Chemotherapy, Shandong Tumor Hospital, Shandong University, Jinan, China; 9 Department of General Surgery, Huashan Hospital Fudan University, Shanghai, China

**Keywords:** PS-341, UBAC2, Obesity, Metabolic inflammation, Insulin resistance

## Abstract

**Background:**

The TM4 (UBAC2) protein, which contains 4 transmembrane domains and one ubiquitin binding domain, is mainly expressed in cell and nuclear membranes. The current research aimed to explore the role of *TM4* in metabolic inflammation and to examine whether the ubiquitin–proteasome inhibitor PS-341 could regulate the function of *TM4*.

**Methods:**

The metabolic phenotypes of *TM4* knockout (KO) mice were studied. We next explored the association between the polymorphisms of *TM4* and obesity in a Chinese Han population. *TM4* expression in the visceral fat of obese patients who underwent laparoscopic cholecystectomy was also analysed. Finally, the effect of PS-341 on the degradation and function of the TM4 protein was investigated in vivo and in vitro.

**Results:**

*TM4* KO mice developed obesity, hepatosteatosis, hypertension, and glucose intolerance under a high-fat diet. TM4 counterregulated Nur77, IKKβ, and NF-kB both in vivo and in vitro. The *TM4* SNP rs147851454 is significantly associated with obesity after adjusting for age and sex (*OR* 1.606, 95% CI 1.065–2.422 *P* = 0.023) in 3394 non-diabetic and 1862 type 2 diabetic adults of Han Chinese. *TM4* was significantly downregulated in the visceral fat of obese patients. PS-341 induced TM4 expression through inhibition of TM4 degradation in vitro. In *db/db* mice, PS-341 administration led to downregulation of Nur77/IKKβ/NF-κB expression in visceral fat and liver, and alleviation of hyperglycaemia, hypertension, and glucose intolerance. The hyperinsulinaemic-euglycaemic clamp demonstrated that PS-341 improved the glucose infusion rate and alleviated insulin resistance in *db/db* mice.

**Conclusions:**

PS-341 alleviates chronic low-grade inflammation and improves insulin sensitivity through inhibition of TM4 degradation.

**Supplementary Information:**

The online version contains supplementary material available at 10.1186/s12986-021-00579-8.

## Introduction

Previous studies have shown that proteasome inhibitors may have anti-inflammatory effects [1–5]. For example, the proteasome inhibitor MG262 inhibited interleukin (IL)-1β/tumour necrosis factor (TNF)-α-induced activation of nuclear factor (NF)-κB in nasal polyp fibroblasts [3]. Reduction of proteasome activity either by partial inhibition with bortezomib or by specifically targeting the immunoproteasome subunit LMP7 limited the secretion of multiple proinflammatory cytokines and chemokines [4]. Chronic renal failure rabbits treated with the proteasome inhibitor MG132 showed decreased NF-κB DNA binding activity and TNF-α expression [1]. Short-term pretreatment with MG-132 reduced TNF-α-induced IL-6 secretion via inhibition of IκBα degradation and the NF-κB pathway in human airway smooth muscle cells [2]. Additionally, ubiquitin–proteasome hyperactivity is associated with enhanced inflammatory reactions and NF-κB expression in diabetic plaques [5].

Obesity is one of the most significant risk factors for multiple nutrition-related disorders, including type 2 diabetes, nonalcoholic fatty liver disease and atherosclerosis [6–8]. Obesity is associated with increased infiltration of macrophages into adipose tissue, causing a cluster of chronic low-grade inflammatory diseases [6]. The efficacy of anti-inflammatory therapy for chronic low-grade inflammatory diseases such as type 2 diabetes and atherosclerosis has been validated in several studies [9, 10]. A clinical trial showed that an interleukin-1-receptor antagonist improved glycaemia and beta-cell secretory function and reduced markers of systemic inflammation in type 2 diabetes [10]. In another study involving 10,061 patients with a history of myocardial infarction, anti-inflammatory therapy targeting the interleukin-1β innate immunity pathway with canakinumab significantly reduced the rate of recurrent cardiovascular events compared to placebo, independent of lipid-levels [9].

The ubiquitin–proteasome pathway is responsible for the degradation of most intracellular proteins in eukaryotes. It may also play a role in the modulation of chronic low-grade inflammation and insulin resistance, and participate in the pathogenesis of metabolic disorders including obesity and type 2 diabetes [11]. However, the relationship between the ubiquitin–proteasome pathway and chronic low-grade inflammatory diseases has not been clearly demonstrated. Our previous work sought to elucidate the pathogenesis of obesity and metabolic inflammation. Tibetan macaques were fed a high-fat diet and injected with low-dose streptozotocin to induce diabetes. Differential gene expression profiles were established in aortic tissues of diabetic Tibetan macaques versus healthy controls using gene chips. An expressed sequence tag (EST), which was identified as GKCFIE07 in our previous study [12], was found to be the most downregulated tag in the aortic tissue of diabetic Tibetan macaques. This EST did not match with any known gene after blasting in dbEST and other databases. Finally, a novel full-length cDNA containing a complete open-reading frame was cloned and analysed using bioinformatic methods reported previously [12]. We provisionally named this novel gene *TM4* since its protein structurally contained four transmembrane domains.

Since *TM4* was the most downregulated gene in the aorta tissue of diabetic Tibetan macaques, we hypothesized that TM*4* might exert an important and protective role against metabolic inflammation. In our previous work, bioinformatics analysis showed that the TM4 (UBAC2) protein contains four transmembrane domains and a ubiquitin binding domain. Hence, we hypothesized that the ubiquitin–proteasome inhibitor PS-341 could alleviate metabolic inflammation through inhibition of TM4 degradation. Ubiquitin–proteasome inhibitors such as PS-341 may serve as a novel therapy for obesity and related disorders, including type 2 diabetes, nonalcoholic fatty liver disease, and atherosclerosis. In the current research, we explored the role of *TM4* in metabolic inflammation and examined whether PS-341 could regulate the degradation and function of TM4 protein both in vitro and in vivo and be a candidate for the treatment of metabolic disorders.

## Methods

### Participants of the TM4 polymorphism study

The present study included 3394 nondiabetic adults and 1862 adults with type 2 diabetes (age ≥ 19) of Han Chinese ancestry who participated in the community-based Shanghai Diabetes Study [13]. The detailed inclusion and exclusion criteria for all subjects have been described previously [13]. The 3394 nondiabetic adults had normal glucose regulation, no family history of diabetes, and were over 40 years old. Normal glucose tolerance was defined as a fasting plasma glucose level < 6.1 mmol/l and a 2-h plasma glucose level of 75 g OGTT < 7.8 mmol/l.

BMI was used to assess generalised obesity according to Chinese criteria, which classified the nondiabetic individuals into two groups: nonobese (BMI < 28 kg/m^2^, n = 2375) and obese (BMI ≥ 28 kg/m^2^, n = 1019).[13, 14] All study participants gave written informed consent, and study protocols were approved by the Institutional Review Board of Shanghai Sixth People’s Hospital, Shanghai Jiaotong University.

### Clinical measurement of the TM4 polymorphism study

All participants underwent detailed clinical investigation as described in a previous publication [13]. In brief, anthropometric parameters, including height, weight, and waist and hip circumference, were measured for all subjects. BMI was calculated as weight in kilograms divided by the square of height in metres, while the waist-to-hip ratio (WHR) was calculated as waist circumference in centimetres divided by hip circumference in centimetres. For the present study, we selected rs147851454 as the tagging single nucleotide polymorphism (SNP) in *TM4*.

### Study on TM4 expression in human visceral fat

Patients who underwent laparoscopic cholecystectomy at Huashan Hospital Fudan University in 2016 were recruited. All participants underwent OGTT except those who had previously been diagnosed with diabetes. In the end, 12 participants were included in the current study. They were divided into 3 groups according to BMI and OGTT: control group (nonobese participants with normal glucose tolerance, *n* = 4), obesity group (obesity with normal glucose tolerance, *n* = 4), and obesity + DM group (obesity with type 2 diabetes, *n* = 4). Adipose tissues were taken from omental tissue during laparoscopic cholecystectomy. All study participants gave written informed consent, and study protocols were approved by the Institutional Review Board of Huashan Hospital Fudan University.

### Mice

*TM4*^*−/−*^ mice were purchased from the Shanghai Research Center for Model Organisms (SRCMO, Shanghai, China), where they were generated by gene trap technology. We obtained 2 heterozygous breeding pairs and used them to generate WT and *TM4-/-* mice, which were then given free access to a high-fat diet (Shanghai SLAC Mouse Diet, containing 16.2% fat by weight). Male *TM4* knockout (KO) mice and wild-type (WT) controls were fed a high-fat diet for 24 weeks. Food intake was measured every day, and weekly weight gain was determined. The metabolic profiles of *TM4* KO and WT mice were documented.

Additionally, weight-matched 4-week-old *db/db* male mice were also obtained from SRCMO and used in the PS-341 intervention experiment. The *db/db* mice were given PS-341 or PBS (intravenously or by gavage, 4 groups) from the age of 5 weeks. Food consumption was recorded daily, and body weight was monitored weekly. All animal procedures were approved by the Institutional Animal Care and Use Committee of Huashan Hospital Fudan University.

The detailed methods of animal phenotype research and molecular biology experiments are provided in the Supplementary materials.

### Statistical analysis

SPSS 17.0 (SPSS, Chicago, IL) was used for statistical analysis. For both in vivo and in vitro studies, values are presented as the mean ± SEM. The results were compared using one-way ANOVA, followed by Tukey–Kramer post hoc test and independent samples *t* test. *P* < 0.05 was considered statistically significant.

For the clinical study, the χ2 test was performed to estimate Hardy–Weinberg equilibrium for each variant before association analysis (Table S1). The allelic and haplotype frequencies were compared using the χ2 test between obese and nonobese subjects. Odds ratios (ORs) with 95% confidence intervals (CIs) were calculated, and associations of SNPs with obesity-related measurements were assessed under a recessive model. A nonparametric approach of rank transformation was taken to assess the results among three genotypes. Unevenly distributed quantitative traits, including BMI, waist circumference, hip circumference, and WHR, were log transformed to approximate normality before analysis.

## Results

### Association between TM4 polymorphism and the risk of obesity

As shown in Table 1, the *TM4* SNP rs147851454 was significantly associated with obesity after adjusting for age and sex (*OR* 1.606, *95% CI* 1.065–2.422 *P* = 0.023).

**Table 1:** Association of *TM4* SNP rs147851454 with obesity

NGT Obesity	CC/CT/TT		C allele frequency		Risk allele (T)		Genotype (unadjusted)		Adjusted with age and gender	
	Over weight	Normal	Cases	Controls	OR (95% CI)	*P*	OR (95%CI)	*P*	OR (95%CI)	*P*
HRM	981/37/1	2320/53/2	0.981	0.988	1.6063 (1.0653,2.4220)	0.0225	1.571 (1.051,2.347)	0.0275	1.547 (1.032,2.319)	0.0347

### TM4 expression in the visceral fat tissue of patients with obesity

**Fig. 1:**
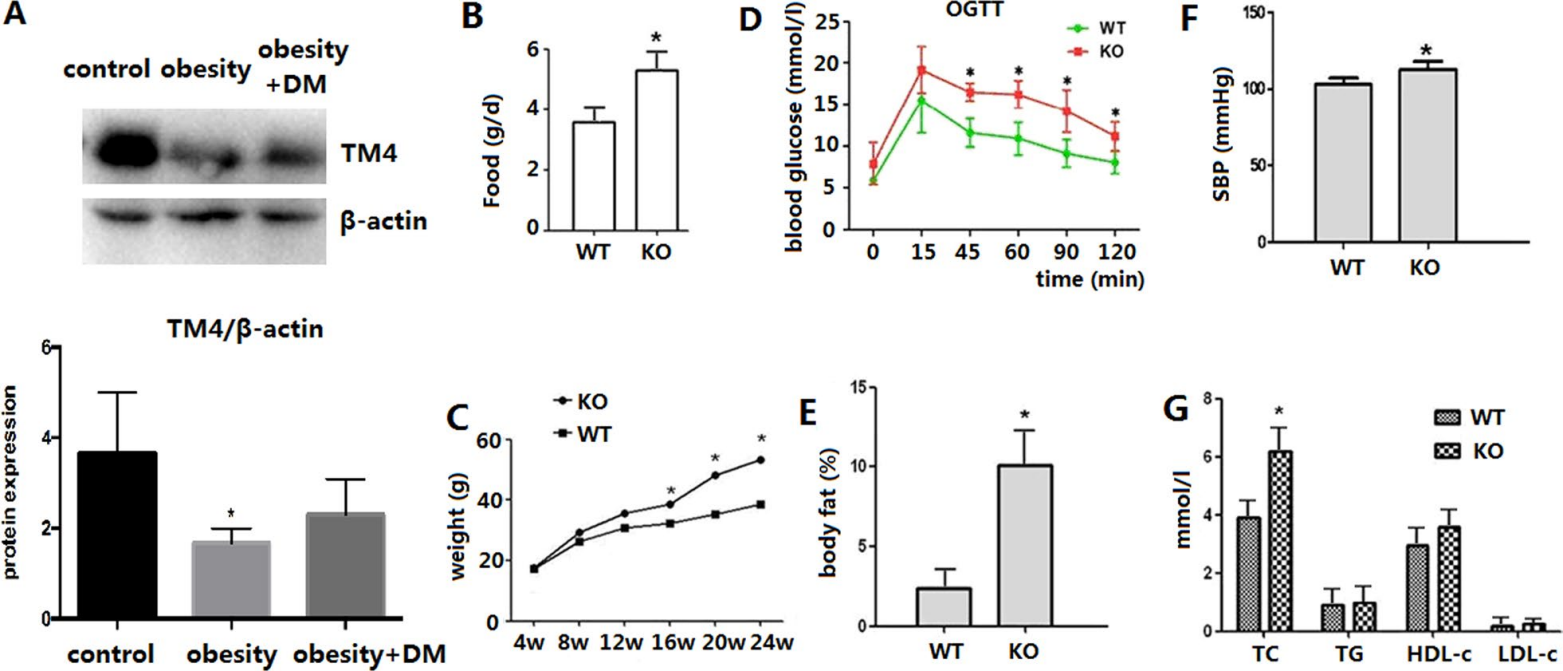
Expression of *TM4* in human visceral fat and the metabolic characteristics of *TM4* knockout mice. **a** *TM4* was significantly downregulated in human visceral fat of patients with obesity when analysed by western blot. **b–g:** Metabolic characteristics of *TM4* knockout mice. **b** Daily food consumption. **c** Weight gain curve. **d** Oral glucose tolerance test at the age of 24 weeks. **e** Body fat content by DEXA. **f** Systolic blood pressure. **g** Serum lipid profiles

*TM4* was significantly downregulated in the visceral fat tissue of patients with obesity when analysed by by western blot (Fig. 1a). *TM4* expression in the visceral fat of the obesity + DM group was also lower than that of the control group. However, the difference was not statistically significant.

### The metabolic phenotypes of the TM4 knockout mice

The expression of TM4 in knockout and wild-type mice was examined by western blot. Additional file 1: Figure S4A shows that *TM4* knockout was successful.

The body weights of male KO mice started to deviate from those of wild-type mice after 3–4 months on a high-fat diet and were 25% higher than WT body weights at the age of 24 weeks (53.2 ± 8.61 vs 38.7 ± 6.22 g, *P* < 0.05, Fig. 1c). Visceral fat mass measured by dual-energy X-ray absorptiometry was markedly increased in male KO mice (10.2 ± 3.32% vs 2.6 ± 0.78%, *P* < 0.05, Fig. 1e, Additional file 1: Figure S4B). *TM4* KO mice also manifested increased food intake (5.24 ± 0.66 vs 3.42 ± 0.38 g, *P* < 0.05, Fig. 1b) and impaired glucose tolerance (Fig. 1d). Compared with WT mice, male KO mice had more severe liver fat deposition (Additional file 1: Figure S3A, S3B, under microscopy; Figure S3C, S3D, under transmission electron microscopy), larger visceral adipocytes (Additional file 1: Figure S3E, S3F) and more macrophage infiltration in visceral adipose tissue (Additional file 1: Figure S3G, S3H). Furthermore, although both groups of mice developed an increased systolic blood pressure (SBP), the SBP elevation of the KO group was greater. The KO mice also exhibited impaired vasodilatation (data not shown).

At the age of 24 weeks, an oral glucose tolerance test (OGTT) was performed. Glucose measurements were taken for fasting levels, as well as 15 min, 45 min, 60 min, 90 min, and 120 min post challenge. The blood glucose levels of *TM4* KO mice at 45 min, 60 min, 90 min, and 120 min post challenge were significantly higher than those of the WT mice (Fig. 1d, all *P* < 0.05).

The mean systolic blood pressure of homozygous *TM4* KO male mice was dramatically higher than that of male WT mice (Fig. 1f, 112.7 ± 5.41 vs. 103.1 ± 4.12 mmHg, *P* = 0.002). The difference in diastolic blood pressure was not significant (74.6 ± 6.25 vs. 72.3 ± 7.81 mmHg, *P* = 0.116).

### Nur77 as an interacting protein of TM4

We identified Nur77, RNF-5, and USP-19 as TM4-interacting proteins by yeast two-hybrid screening.

**Fig. 2:**
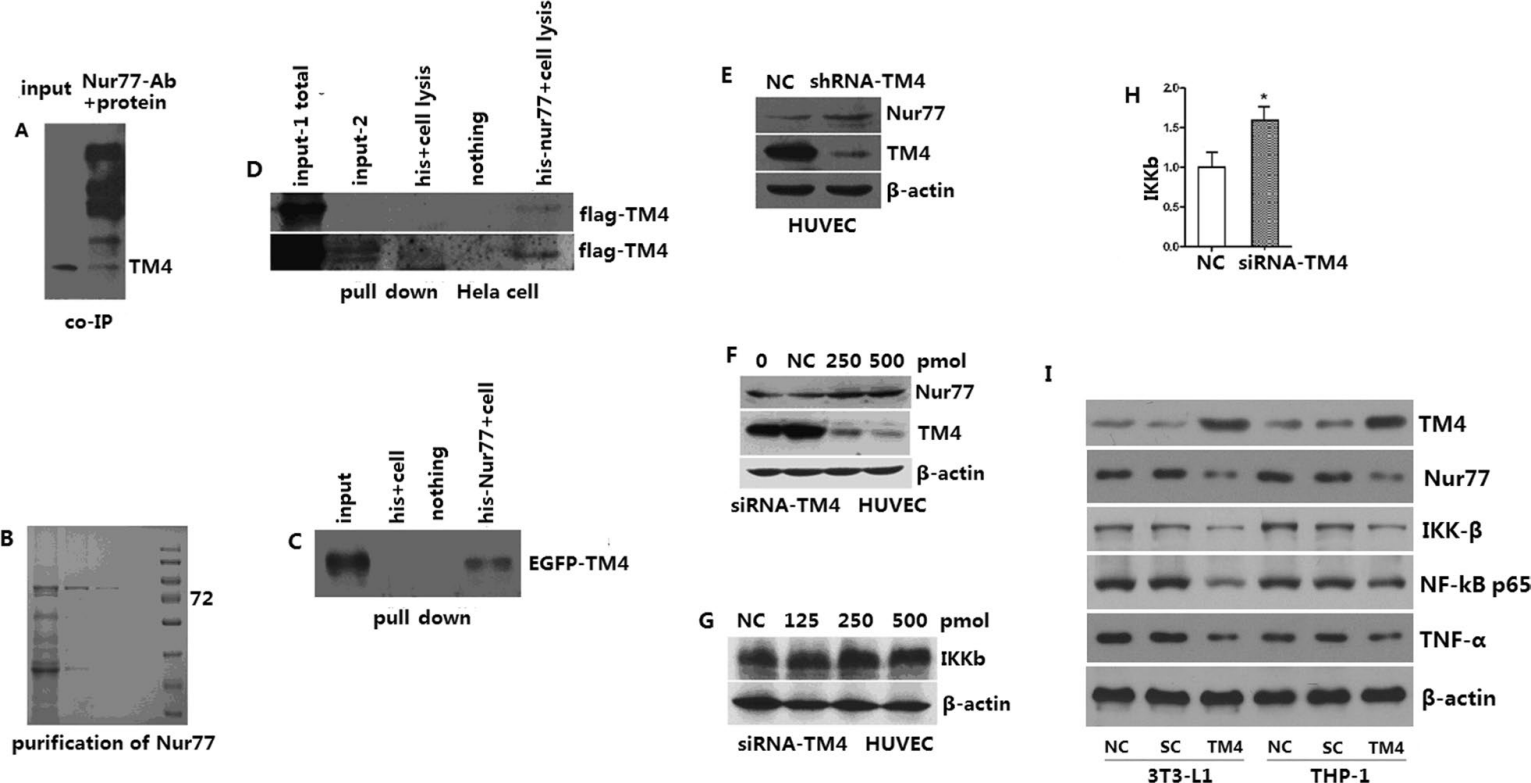
Coimmunoprecipitation of Nur77 and TM4 and pulldown. **a** Coimmunoprecipitation of Nur77 and TM4. **b** Nur77 expression and purification. **c** Pulldown. **d** Pull down (HeLa cell). **e** TM4 blockade with lentivirus and its effect on the expression of Nur77. **f** TM4 interference and its effect on the expression of Nur77. **g** TM4 interference and its effect on the expression of IKK-β (western blot). **h** TM4 interference and its effect on the expression of IKK-β (real-time PCR). **i** TM4 counterregulates Nur77, IKKβ, and NF-κB

The eukaryotic expression plasmid for Nur77 was transfected into Hela cells using Lipofectamine 2000 to induce Nur77 overexpression. Cells were harvested 48 h later, lysed with IP lysis buffer, and incubated with Nur77 antibodies. Protein A/G beads were then applied to obtain a compound body. After elution and purification, the protein obtained was further examined by western blot analysis. TM4 was present (Fig. 2a) in the group using Nur77 antibodies for specific precipitation.

The TM4/Nur77 interaction was verified with a pulldown assay (Fig. 2b, c). To circumvent issues associated with a self-prepared antibody, such as low titer, we constructed eukaryotic EGFP-C2-TM4, Flag-TM4, and other plasmids, confirmed by sequencing and cell expression. First, EGFP-C2-TM4 and Flag-TM4 were overexpressed in eukaryotic cells. Forty-eight hours later, the cells were harvested, lysed, incubated with the recombinant Nur77 protein obtained from prokaryotic expression, eluted and purified. The product was examined by SDS-PAGE electrophoresis using GFP and flag antibodies (Fig. 2d).

### *TM4 counterregulates Nur77, IKKβ, and NF-κB: *in vitro* studies*

The TM4 lentiviral vector was packaged and verified, and then the changes in the expression levels of the interacting protein Nur77 were examined through western blot after TM4 interference (Fig. 2e). TM4 interference increased Nur77 protein expression (Fig. 2f), and the regulation was not at the transcriptional level. Additionally, TM4 interference with siRNA increased IKK-β expression (Fig. 2g, h), and this effect was dose-dependent (Fig. 2g). Moreover, TM4 interference with siRNA increased NF-κB expression (data not shown). As shown, the expression of Nur77, IKKβ, and NF-κB was downregulated after TM4 overexpression in both 3T3-L1 and THP-1 cells (Fig. 2i).

### The ubiquitin–proteasome inhibitor PS-341 inhibits the degradation of TM4

**Fig. 3:**
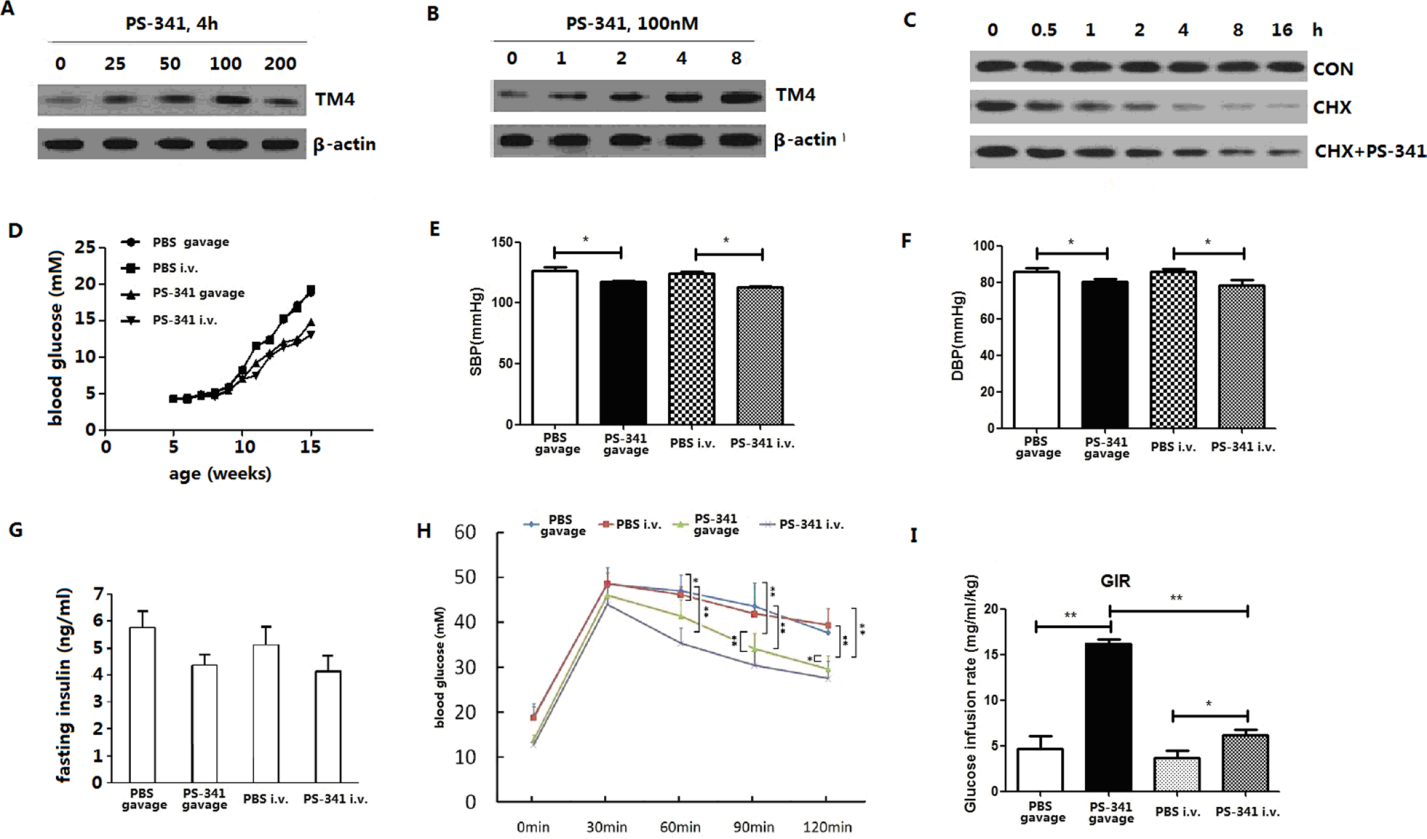
Effect of PS-341 on TM4 protein and metabolic homeostasis. **a, b** The effect of PS-341 on TM4 expression in HUVECs. **c** PS-341 retards TM4 degradation in HUVEC. **d** Blood glucose of *db/db* mice treated with PS-341. **e** SBP of *db/db* mice treated with PS-341. **f** DBP of *db/db* mice treated with PS-341. **g** Fasting insulin of *db/db* mice treated with PS-341. **h** Oral glucose tolerance test. **i** Glucose infusion rate after glucose homeostasis

Administration of PS-341 inhibited TM4 protein degradation in HUVECs in a concentration-dependent (Fig. 3a) and time-dependent (Fig. 3b) manner. The maximal effect of PS-341 was observed at 100 nM.

After HUVECs were treated with CHX, TM4 protein expression decreased in a time-dependent manner. Moreover, PS-341 inhibited TM4 degradation (Fig. 3c). The half-life of the TM4 protein was 1 h, and it extended to 4 h after PS-341 intervention (Table S2).

### The effect of PS-341 on db/db mice

Fasting blood glucose was measured every week (Fig. 3d). Blood sugar gradually rose with age, with a rapid increase after 10 weeks. The differences in glucose became statistically evident at the age of 11 weeks, with the fasting glucose of the PS-341 gavage group being significantly lower than that of the PBS gavage group (9.2 ± 0.94 vs 11.5 ± 0.63, *P* < 0.005). Furthermore, the fasting glucose of the PS-341 intravenous (iv) group was significantly lower than that of the PBS intravenous group (7.5 ± 0.99 vs 11.6 ± 0.82, *P* < 0.005), and the fasting glucose of the PS-341 intravenous group was lower than that of the PS-341 gavage group (*P* < 0.05). At the age of 15 weeks, the fasting glucose of both PS-341 groups was lower than that of the PBS control groups (14.9 ± 0.91 vs 18.8 ± 1.90, 13.1 ± 1.19 vs 19.4 ± 2.29, *P* < 0.005), and the fasting glucose of the PS-341 intravenous group was still lower than that of the PS-341 gavage group (*P* < 0.05).

Tail artery blood pressure was measured every week (Fig. 3e, f), and at the age of 15 weeks, systolic pressure (SBP) and diastolic blood pressure (DBP) were compared. The levels of both SBP and DBP in the PS-341 gavage group were significantly lower than those in the PBS gavage group (118 ± 3.6 vs 125 ± 2.5, 80 ± 1.7 vs 86 ± 2.1, *P* < 0.005). Moreover, both SBP and DBP of the PS-341 iv group were lower than those of the PBS iv group (112 ± 4.1 vs 124 ± 1.0, 78 ± 3.1 vs 86 ± 1.5, *P* < 0.005).

Fasting insulin levels were also measured at the age of 15 weeks. The results showed a significantly lower fasting insulin level for the PS-341-treated group than for the PBS control group (Fig. 3g).

Fasting blood glucose (0 min) was also determined, and blood glucose was measured 30 min, 60 min, 90 min, and 120 min after 10% glucose solution gavage in *db/db* mice. The 60-min, 90-min, and 120-min blood glucose levels of PS-341-treated mice were significantly lower than those of the PBS controls, and the blood glucose level of the PS-341 iv group was lower than that of the PS-341 gavage group (Fig. 3h).

During the hyperinsulinaemic-euglycaemic clamp test, the glucose infusion rate (GIR) at 60 ~ 120 min was analysed (Fig. 3i). The GIR of the PS-341 gavage group was significantly higher than that of the PBS gavage group (*P* < 0.05), the GIR of the PS-341 iv group was significantly higher than that of the PBS iv group (*P* < 0.05), and the GIR of PS-341 gavage group was significantly higher than that of the PS-341 iv group (*P* < 0.05).

**Fig. 4:**
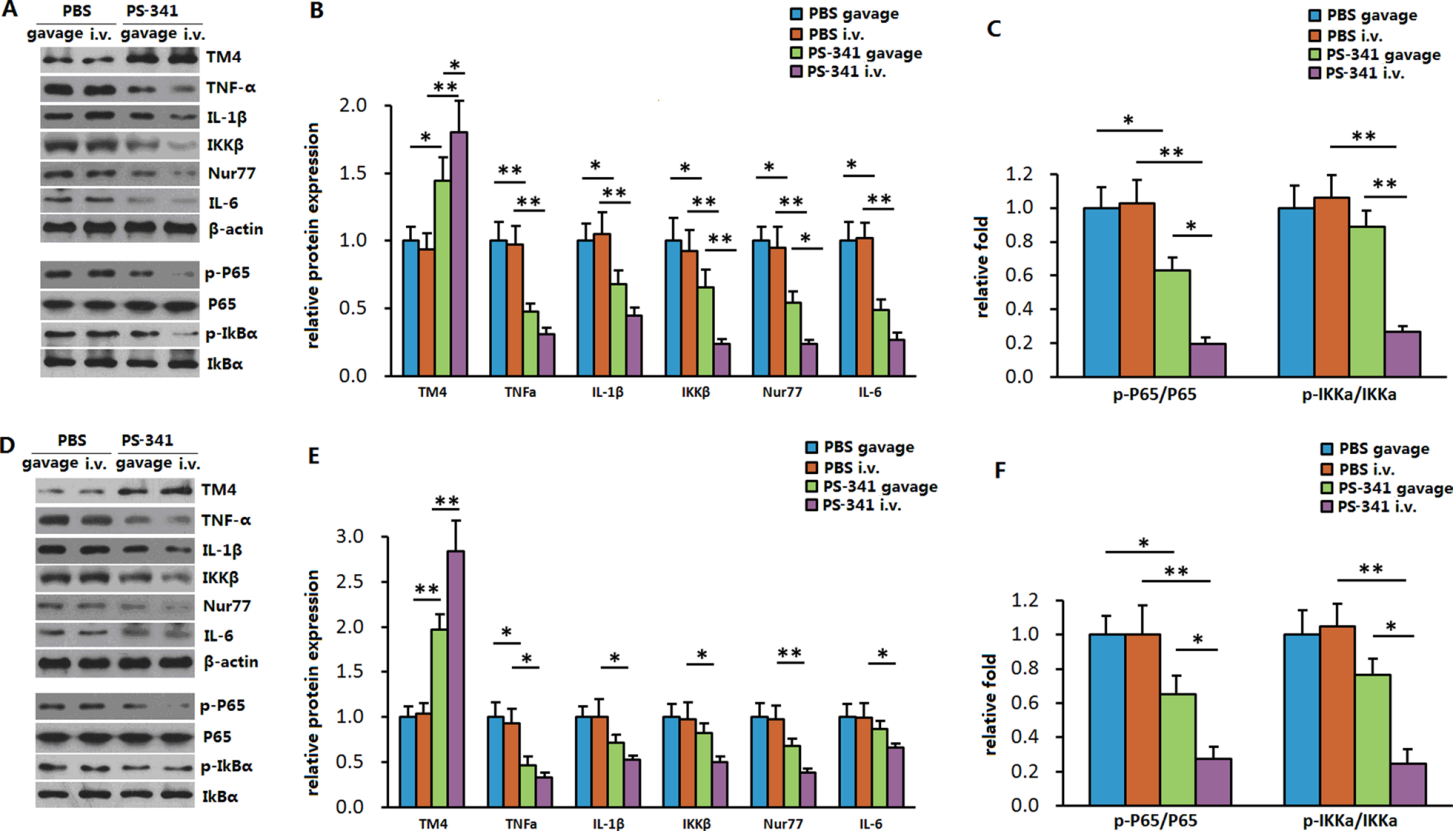
Expression of TM4 and proinflammatory genes after PS-341 administration. **a** western blot of TM4 and proinflammatory genes in the liver tissue of *db/db* mice. **b** The relative fold expression of TM4 and proinflammatory genes in the liver tissue of *db/db* mice. **c** The relative fold phosphorylation of IkB-α and P65 in the liver tissue of *db/db* mice. **d** western blot of TM4 and proinflammatory genes in the epididymal fat tissue of *db/db* mice. **e** The relative fold expression of TM4 and proinflammatory genes in the epididymal fat tissue of *db/db* mice. **f** The relative fold phosphorylation of IkB-α and P65 in the epididymal fat tissue of *db/db* mice

After administration of PS-341, TM4 expression was induced in the liver (Fig. 4a) and epididymal fat (Fig. 4d) tissues of *db/db* mice, while the expression of the proinflammatory adipokines TNF-α, IL-1β, and IL-6 (Fig. 4b, e) and phosphorylation of IkB-α and P65 (Fig. 4c, f) were downregulated significantly.

## Discussion

TM4 (UBAC2) is a protein belonging to the UBA (ubiquitin-binding domain) family and contains a highly conserved functional UBA domain in the C-terminus [15]. The structure of TM4 is highly conserved in different species, and it is ubiquitously expressed in various tissues, including the bone marrow, spleen, heart, liver, brain, pancreas, kidney, lung, muscle, and fat. One important function of UBA family proteins is related to ubiquitin-mediated protein degradation. They may interact specifically with certain ubiquitinated proteins, which are then transported to the proteasomes for degradation [16].

In the clinical part of the current study, *TM4* SNP rs147851454 was significantly associated with obesity in a large sample of Han Chinese adults. *TM4* was significantly downregulated in the visceral fat of obese patients who underwent laparoscopic cholecystectomy. Furthermore, our in vitro research demonstrated that TM4 expression was significantly downregulated by free fatty acids (Additional file 1: Figure S5C). Through yeast two-hybrid screening, we found that Nur77 was one of the interacting proteins of TM4 and further confirmed it by coimmunoprecipitation and pulldown assays. As an important member of the orphan nuclear receptor family, Nur77 is involved in various intracellular signal transduction pathways. Nur77 can directly regulate the expression of IKKβ and is thereby engaged in the signal transduction of the NF-κB inflammatory pathway. [17, 18] In addition, Nur77 attenuates AMPK activation by binding with liver kinase B1 (LKB1) [19].

Our animal study showed that the expression of Nur77 and IKKβ was significantly upregulated after *TM4* knockout. Moreover, obesity developed earlier, and weight gain was more significant in *TM4* KO mice than in wild-type mice on a high-fat diet. The underlying mechanism was deduced as follows: a high-fat diet leads to TM4 downregulation, thereby enhancing the expression of Nur77 and IKKβ, which will subsequently activate the NF-κB pathway and induce inflammatory cytokine secretion.

PS-341 is a proteasome inhibitor with boronic acid peptide chemical properties. In our in vitro study, PS-341 administration did not alter TM4 mRNA synthesis significantly, but dramatically increased TM4 protein expression, indicating that PS-341 inhibited the degradation of TM4 protein. Cycloheximide (CHX) is commonly used to determine the half-life of proteins in molecular biology studies. In our study, cells were first treated with CHX. Then, western blotting was performed to determine TM4 protein changes over time.

Since CHX inhibits TM4 synthesis, intracellular TM4 will decline. Meanwhile, PS-341 administration may delay the TM4 decrease due to CHX treatment, indicating that the ubiquitin–proteasome inhibitor PS-341 has extended the half-life of TM4, possibly through the inhibition of TM4 degradation. Hence, PS-341 might alleviate chronic low-grade inflammation by inhibiting TM4 degradation.

**Fig. 5:**
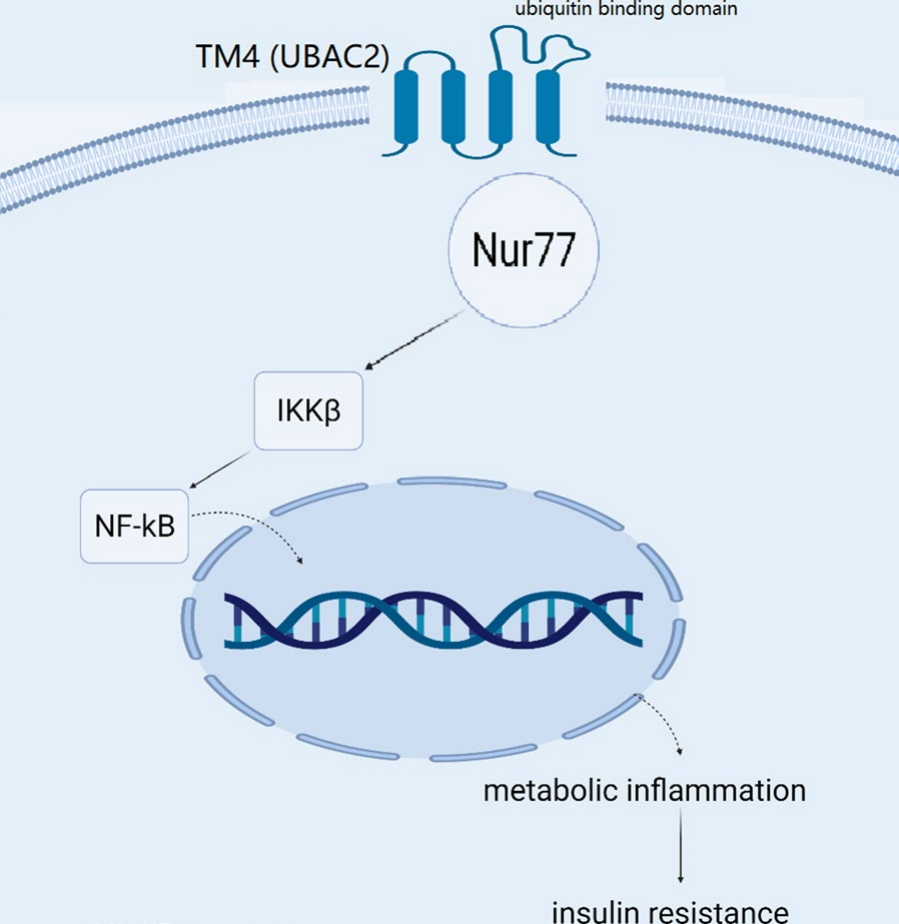
A graphic summary depicting the effect of PS-341 and TM4 in chronic low-grade inflammation and insulin sensitivity

After treatment with PS-341, TM4 expression in the epididymal fat and liver of *db/db* mice was significantly increased, while the expression of Nur77, IKKβ, IκB, and NF-κB was downregulated, indicating that PS-341 inhibited chronic metabolic inflammation through the TM4-Nur77-IKKβ-NF-κB pathway (Fig. 5), thus improving blood glucose, glucose tolerance, blood pressure, and other metabolic parameters. In our study, the hyperinsulinaemic-euglycaemic clamp test was performed, and the glucose infusion rate (GIR) was used to evaluate insulin sensitivity. As shown above, the GIR of the PS-341 group was clearly higher than that of the PBS control, indicating that PS-341 was able to improve insulin sensitivity through TM4.

PS-341 is an anticancer drug and the first therapeutic proteasome inhibitor to be clinically used in humans [20]. It has been approved in the U.S. and Europe for treating multiple myeloma and mantle cell lymphoma [20]. Our current study reveals that PS-341 alleviates chronic low-grade inflammation and insulin resistance through inhibition of TM4 degradation, which validates a previous hypothesis that blood cancers and chronic metabolic diseases share a common pathogenic mechanism: aberrant inflammatory responses [21].

In the current study, *TM4* global knockout mice were used. *TM4* KO mice are characterized by IKKβ/NF-κB pathway activation and insulin resistance. *TM4* KO mice developed obesity, hepatosteatosis, hypertension, and glucose intolerance on a high-fat diet. According to a previous study, hepatic IKKβ/NF-kB pathway activation leads to insulin resistance both locally in the liver and systemically [22]. However, NF-kB inhibition by p65-KO in fat tissue failed to reduce fat inflammation and improve insulin sensitivity in obese mice, although inhibition was observed in lean mice [23]. In our future research, adipose- or liver-specific *TM4* knockout mice will be developed and studied in-depth.

In light of the well-established concept of obesity and its related metabolic disorders being a cluster of chronic low-grade inflammatory diseases,[6–8] we proposed the concept of metabolic inflammatory syndrome (MIS), with obesity, type 2 diabetes, nonalcoholic fatty liver disease, and atherosclerosis as its 4 components.[24, 25] Our previous study verified that miR-145 alleviates MIS through multiple pathways.[26, 27] It is possible that PS-341, as an inhibitor of low-grade inflammation, may serve as a therapeutic agent for MIS. Future studies should explore whether interactions between TM4 and Nur77 are regulated by glucose levels and/or inflammatory factors and whether the posttranslational modifications of Nur77 are altered in MIS using proteomics and molecular imaging techniques. In addition, it would be of great interest to delineate the spatiotemporal dynamics of TM4/Nur77 in response to PS-341 using patient-derived organoids and high-resolution imaging techniques [28, 29].

## Conclusions

In conclusion, the proteasome inhibitor PS-341 alleviates chronic low-grade inflammation while improving insulin sensitivity through upregulation of TM4 expression and may be a candidate for the treatment of metabolic inflammatory diseases, such as obesity and type 2 diabetes.

## Supplementary Information

**Additional file 1.** Supplementary methods and results.

## Data Availability

The datasets analysed during the current study are available from the corresponding author on reasonable request.

## Abbreviations

EST: Expressed sequence tag
OGTT: Oral glucose tolerance test
BMI: Body mass index
WHR: Waist-to-hip ratio
SNP: Single nucleotide polymorphism
MIS: Metabolic inflammatory syndrome

## References

[1] Feng B, Zhang Y, Mu J, Ye Z, Zeng W, Qi W, Luo Z, Guo Y, Yang X, Yuan F (2010). Preventive effect of a proteasome inhibitor on the formation of accelerated atherosclerosis in rabbits with uremia. J Cardiovasc Pharmacol.

[2] Moutzouris JP, Che W, Ramsay EE, Manetsch M, Alkhouri H, Bjorkman AM, Schuster F, Ge Q, Ammit AJ (2010). Proteasomal inhibition upregulates the endogenous MAPK deactivator MKP-1 in human airway smooth muscle: mechanism of action and effect on cytokine secretion. Biochim Biophys Acta.

[3] Pujols L, Fernandez-Bertolin L, Fuentes-Prado M, Alobid I, Roca-Ferrer J, Agell N, Mullol J, Picado C (2012). Proteasome inhibition reduces proliferation, collagen expression, and inflammatory cytokine production in nasal mucosa and polyp fibroblasts. J Pharmacol Exp Ther.

[4] Schmidt N, Gonzalez E, Visekruna A, Kuhl AA, Loddenkemper C, Mollenkopf H, Kaufmann SH, Steinhoff U, Joeris T (2010). Targeting the proteasome: partial inhibition of the proteasome by bortezomib or deletion of the immunosubunit LMP7 attenuates experimental colitis. Gut.

[5] Marfella R, D'Amico M, Esposito K, Baldi A, Di Filippo C, Siniscalchi M, Sasso FC, Portoghese M, Cirillo F, Cacciapuoti F, Carbonara O, Crescenzi B, Baldi F, Ceriello A, Nicoletti GF, D'Andrea F, Verza M, Coppola L, Rossi F, Giugliano D (2006). The ubiquitin-proteasome system and inflammatory activity in diabetic atherosclerotic plaques: effects of rosiglitazone treatment. Diabetes.

[6] Sell H, Habich C, Eckel J (2012). Adaptive immunity in obesity and insulin resistance. Nat Rev Endocrinol.

[7] Ross R (1999). Atherosclerosis–an inflammatory disease. N Engl J Med.

[8] Hotamisligil GS (2017). Inflammation, metaflammation and immunometabolic disorders. Nature.

[9] Ridker PM, Everett BM, Thuren T, MacFadyen JG, Chang WH, Ballantyne C, Fonseca F, Nicolau J, Koenig W, Anker SD, Kastelein J, Cornel JH, Pais P, Pella D, Genest J, Cifkova R, Lorenzatti A, Forster T, Kobalava Z, Vida-Simiti L, Flather M, Shimokawa H, Ogawa H, Dellborg M, Rossi P, Troquay R, Libby P, Glynn RJ (2017). Antiinflammatory therapy with canakinumab for atherosclerotic disease. N Engl J Med.

[10] Larsen CM, Faulenbach M, Vaag A, Volund A, Ehses JA, Seifert B, Mandrup-Poulsen T, Donath MY (2007). Interleukin-1-receptor antagonist in type 2 diabetes mellitus. N Engl J Med.

[11] Wing SS (2008). The UPS in diabetes and obesity. BMC Biochem.

[12] Hu RM, Han ZG, Song HD, Peng YD, Huang QH, Ren SX, Gu YJ, Huang CH, Li YB, Jiang CL, Fu G, Zhang QH, Gu BW, Dai M, Mao YF, Gao GF, Rong R, Ye M, Zhou J, Xu SH, Gu J, Shi JX, Jin WR, Zhang CK, Wu TM, Huang GY, Chen Z, Chen MD, Chen JL (2000). Gene expression profiling in the human hypothalamus-pituitary-adrenal axis and full-length cDNA cloning. Proc Natl Acad Sci USA.

[13] Hu C, Wang C, Zhang R, Ng MC, Bao Y, Wang C, So WY, Ma RC, Ma X, Chan JC, Xiang K, Jia W (2010). Association of genetic variants of NOS1AP with type 2 diabetes in a Chinese population. Diabetologia.

[14] Zhou BF (2002). Predictive values of body mass index and waist circumference for risk factors of certain related diseases in Chinese adults–study on optimal cut-off points of body mass index and waist circumference in Chinese adults. Biomed Environ Sci.

[15] Sawalha AH, Hughes T, Nadig A, Yilmaz V, Aksu K, Keser G, Cefle A, Yazici A, Ergen A, Alarcon-Riquelme ME, Salvarani C, Casali B, Direskeneli H, Saruhan-Direskeneli G (2011). A putative functional variant within the UBAC2 gene is associated with increased risk of Behcet's disease. Arthritis Rheum.

[16] Madura K (2002). The ubiquitin-associated (UBA) domain: on the path from prudence to prurience. Cell Cycle.

[17] Pei L, Castrillo A, Chen M, Hoffmann A, Tontonoz P (2005). Induction of NR4A orphan nuclear receptor expression in macrophages in response to inflammatory stimuli. J Biol Chem.

[18] Pei L, Castrillo A, Tontonoz P (2006). Regulation of macrophage inflammatory gene expression by the orphan nuclear receptor Nur77. Mol Endocrinol.

[19] Zhan YY, Chen Y, Zhang Q, Zhuang JJ, Tian M, Chen HZ, Zhang LR, Zhang HK, He JP, Wang WJ, Wu R, Wang Y, Shi C, Yang K, Li AZ, Xin YZ, Li TY, Yang JY, Zheng ZH, Yu CD, Lin SC, Chang C, Huang PQ, Lin T, Wu Q (2012). The orphan nuclear receptor Nur77 regulates LKB1 localization and activates AMPK. Nat Chem Biol.

[20] Dou QP, Zonder JA (2014). Overview of proteasome inhibitor-based anti-cancer therapies: perspective on bortezomib and second generation proteasome inhibitors versus future generation inhibitors of ubiquitin-proteasome system. Curr Cancer Drug Targets.

[21] Tall AR, Levine RL (2017). Cardiovascular disease: commonality with cancer. Nature.

[22] Cai D, Yuan M, Frantz DF, Melendez PA, Hansen L, Lee J, Shoelson SE (2005). Local and systemic insulin resistance resulting from hepatic activation of IKK-beta and NF-kappaB. Nat Med.

[23] Gao Z, Zhang J, Henagan TM, Lee JH, Ye X, Wang H, Ye J (2015). P65 inactivation in adipocytes and macrophages attenuates adipose inflammatory response in lean but not in obese mice. Am J Physiol Endocrinol Metab.

[24] Hu R, Xie Y, Lu B, Chen F, Li L, Huang Y, Li Q, Ye W, Zhang Z, Zhou L, He M, Fan W, Liu J, Wen J, Chen L, Yang Y, Li Y, Zhu X (2016). High detective rate of "metabolic inflammatory syndrome" in patients with type 2 diabetes. Chin J Endocrinol Metabol.

[25] Hu R, Xie Y, Lu B, Li Q, Chen F, Li L, Hu J, Huang Y, Li Q, Ye W, Li R, Liu N, Huang J, Zhang Z, Zhou L, He M, Fan W, Liu J, Wen J, Chen L, Yang Y, Li Y, Fan D, Zhu X (2018). Metabolic inflammatory syndrome: a novel concept of holistic integrative medicine for management of metabolic diseases. AME Med J.

[26] Li R, Shen Q, Wu N, He M, Liu N, Huang J, Lu B, Yao Q, Yang Y, Hu R (2018). MiR-145 improves macrophage-mediated inflammation through targeting Arf6. Endocrine.

[27] He M, Wu N, Leong MC, Zhang W, Ye Z, Li R, Huang J, Zhang Z, Li L, Yao X, Zhou W, Liu N, Yang Z, Dong X, Li Y, Chen L, Li Q, Wang X, Wen J, Zhao X, Lu B, Yang Y, Wang Q, Hu R (2019). miR-145 improves metabolic inflammatory disease through multiple pathways. J Mol Cell Biol.

[28] Yao X, Smolka AJ (2019). Gastric parietal cell physiology and helicobacter pylori-induced disease. Gastroenterology.

[29] Liu X, Xu L, Li J, Yao PY, Wang W, Ismail H, Wang H, Liao B, Yang Z, Ward T, Ruan K, Zhang J, Wu Q, He P, Ding X, Wang D, Fu C, Dou Z, Wang W, Liu X, Yao X (2020). Mitotic motor CENP-E cooperates with PRC1 in temporal control of central spindle assembly. J Mol Cell Biol.

