# Supplementary material for: PS-341 alleviates chronic low-grade inflammation and improves insulin sensitivity through the inhibition of TM4 (UBAC2) degradation
Source: Nutr Metab (Lond). 2021 Jun 1;18:54. doi: 10.1186/s12986-021-00579-8 (PMC8170790; doi:10.1186/s12986-021-00579-8)
Supplement: Supplementary file 1 — Additional file 1. Supplementary methods and results. [file 12986_2021_579_MOESM1_ESM.docx]

**Supplementary materials**

**Methods**

**Metabolic phenotype study of mice**

At 24 weeks of age, the mice were anaesthetized with an intraperitoneal injection of ketamin-xylazine mixture (100 mg/kg ketamine and 10 mg/kg xylazine), and their body fat was measured using dual energy X-ray absorbitometry (DEXA). For glucose tolerance tests, both 24-week-old male wild-type (n=9) and *TM4^-/-^* (n=10) mice were fasted overnight (14 hours) and then fed a 2 g/kg glucose solution by gavage. Glucose levels were measured for the fasting state and then 15 minutes, 45 minutes, 60 minutes, 90 minutes, and 120 minutes post challenge. Blood samples were collected from the tail vein at the indicated time, and glucose was measured using an Ascensia glucometer.

Twenty-four-week-old male mice were euthanised under tribromoethanol anaesthesia. Tissue lysates were prepared and used for western blotting. Tissues including liver and epididymis fat were removed and immediately frozen in liquid nitrogen.

**Determination of mouse tail blood pressures**

A computer-assisted, noninvasive, tail artery blood pressure monitor (BP-98A, Softron Biotechnology Incorporated, China) was used for the following measurement and analysis. In a quiet environment, the mice were placed in a bag with a preheated heating tube to maintain a constant temperature for vasodilation of the tail vessels. Blood pressure data were recorded via a computer data acquisition software system. The blood pressure of each mouse was measured 3-5 times, and the mean value was calculated. This was repeated three times.

The difference in tail artery blood pressure between *TM*4^-/-^ and control mice was further verified in a double-blinded test by Professor Youfei Guan of Peking University.

**Hyperinsulinaemic-euglycaemic clamp studies**

The right jugular veins of the *db/db* mice were catheterized for the hyperinsulinaemic-euglycaemic clamp test. Insulin was infused continuously, and blood glucose levels were measured every 5 minutes. After the blood glucose of the mice in each group stabilized, a 10% glucose solution was infused to maintain blood glucose homeostasis based on individual blood glucose levels.

**Oral Glucose Tolerance Test (OGTT)**

Glucose tolerance was assessed by OGTT after mice were fasted for 12-16 hours. A glucose solution (2 g/kg) bolus was administered by gavage. Blood samples were collected from the tail vein at 0, 15, 30, 60 and 120 minutes after administration, and glucose was measured using a One-Touch Glucometer (LifeScan, Milpitas, CA). The mean area under the OGTT curve (AUC) was calculated.

**Histological and Morphometric Analyses**

Samples were taken from the liver and epididymal adipose tissue. Each tissue sample was cut into 2 pieces, fixed in paraformaldehyde (4%), embedded in paraffin, and cut into 5 µm sections. The average size of adipocytes was measured with images captured from haematoxylin and eosin-stained sections. Each area was assessed under 400× magnification within the adipose tissue, and a mean was obtained by quantitative morphometry with automated image analysis (Image-Pro Plus, Version 5.0; Media Cybernatics, Houston, TX).

**Immunohistochemical Staining**

Paraffin sections were processed for immunohistochemistry using a microwave-based antigen retrieval method. The sections were incubated with primary rat polyclonal anti-F4/80 antibody (Abcam, Cambridge, MA, USA) overnight and then with a matching biotinylated secondary antibody for 30 minutes at 37°C. The primary antibody was omitted as a negative control, and the stained sections were developed with diaminobenzidine and counterstained with haematoxylin. The results were viewed under an optic microscope (Olympus, Japan).

**Cell culture**

3T3-L1 preadipocytes were maintained in Dulbecco's modified Eagle's medium (DMEM) supplemented with 10% calf serum. To differentiate, cells were grown to confluence, placed into induction medium (DMEM, 10% cosmic calf serum, 5 μg/ml insulin, 0.5 mM IBMX, 1 μM dexamethazone, and 10 μM TZD) for 2 days, and then switched into differentiation medium (DMEM, 10% cosmic calf serum, 5 μg/ml insulin) until ready for experimentation. For RNAi experiments, siRNA specific to TM4 (target sequence: GCAGUAGUCUGCUUAUUUATT) and a nonspecific control were synthesized by Qiagen. Amaxa Nucleofector was used to electroporate siRNA into 3T3-L1 adipocytes 5 days after inducing differentiation, and experiments were performed 36-48 hours after electroporation. Cells were seeded in 6-well plates, grown to ~50% confluency and transfected with plasmid DNA.

**Quantitative PCR**

RNA was isolated from tissues and cells using TRIzol (Invitrogen), and cDNA was synthesized using either ThermoScript (Invitrogen) or iScript (BioRad). Q-PCR was performed using a BioRad iCycler. The forward primer sequence of IKKβ was 5’-AGAAGGAGCGGCTACTGGA-3’. The reverse primer sequence of IKKβ was 5’-ATTTCTGGCTGGTTGGTGAT-3’.

**Yeast two-hybrid system**

The yeast two-hybrid system was used to screen the candidate interaction partners of the TM4 protein. The open reading frame of the TM4 gene was amplified and cloned into the pGB vector to construct a pGB-TM4 recombinant yeast plasmid. The pGB-TM4 recombinant plasmid was then used as bait to test whether it can induce the expression of the reporter gene to self-activate. The yeast strain Y190 was transfected with the pGB-TM4 recombinant plasmid to test whether the bait gene was able to activate the expression of the reporter gene. Afterwards, the yeast cDNA library was screened, positive clones were identified, and the yeast plasmid was extracted. Prey and bait plasmids were cotransfected into the yeast Y190 strain to further examine the interaction between the prey and bait proteins. Finally, the positive plasmids were sequenced, and the Blastn program was used to characterize the sequence.

**Immunoprecipitation and pull down**

The interaction between TM4 and Nur77 was verified by immunoprecipitation and pulldown assays. First, the Nur77 overexpression plasmid was constructed and transfected into HeLa cells, which were then harvested and lysed with IP lysis buffer. A Nur77-specific antibody was used to capture the complex, and protein A/G was used to precipitate the complex. Then, western blotting was performed for analysis. Next, Nur77 was overexpressed in BL21 bacteria, and specific beads were used to purify and harvest the protein. Flag-TM4 was constructed, and EGFP-TM4 overexpression plasmids were transfected into cells. The cell lysis product was then collected, mixed with Nur77 protein, gently rotated at 4°C, and pulled down by beads. Western blot was performed for verification.

**Western blot**

Treated cells were washed in ice-cold PBS and extracted for 30 minutes with RIPA buffer containing a cocktail of 10% phosphatase inhibitor and 10% protease inhibitor (Sigma, St. Louis, MO). Lysates were cleared by centrifugation at 13,000 × g for 30 minutes, and protein concentrations were determined using a Bio-red protein assay. Equivalent amounts of whole cell extracts were resolved by SDS-polyacrylamide gel electrophoresis, transferred onto a polyvinylidene difluoride membrane (Millipore Corporation, MA, USA), and then probed with specific antibodies. Immune complexes were detected using horseradish peroxidase conjugated with either anti-mouse or anti-rabbit antibodies, followed by chemiluminescence detection (Perkin Elmer Cetus, Foster City, CA, USA). We used antibodies against TM4 (UBAC2, Sigma Group Inc., USA), Nur77 (Santa Cruz), IKKβ (Cell Signaling Technology), and NF-κB (Cell Signaling Technology), followed by an anti-IgG horseradish peroxidase-conjugated secondary antibody. The protein level was normalized to that of β-actin, an internal control, and phosphorylated protein was normalized to that of total protein.

**Results**

**The expression profiles and cell location of TM4 (UBAC2) protein**

The human *TM4* gene was mapped on chromosome 13q32.3 and had nine exons that encoded a protein of 344 amino acids (Figure S1A). Online bioinformatics analysis showed that there were four transmembrane helixes in the TM4 protein that spanned amino acids 20 to 42, 92 to 114, 129 to 151, and 164 to 186 (<http://www.cbs.dtu.dk/services/TMHMM/>) (Figure S1A, S1B). There was also a ubiquitin-associated domain in the C-terminus of the protein (<https://www.ncbi.nlm.nih.gov/Structure/cdd/wrpsb.cgi>) (Figure S1A, 1C).

Bioinformatics analysis showed that the TM4 protein contains 4 transmembrane domains and a ubiquitin-binding domain (Figure S2A). As depicted in Figure S2B, TM4 is expressed in various tissues, including the aorta, adipose layers, liver, muscle, pancreas, etc.

**Lipid profiles of *TM4* KO mice after high fat diet**

After receiving a high-fat diet, the weights of *TM4* KO mice were significantly higher than those of the WT controls. We further examined the lipid profiles and found that the serum total cholesterol (TC) of *TM4* KO mice was significantly higher than that of WT controls (6.19 vs. 3.91 mmol/L, *P*＜0.05). The levels of serum triglyceride (TG), high-density lipoprotein cholesterol (HDL-c), and low-density lipoprotein cholesterol (LDL-c) in TM4 KO mice were also higher than those in WT mice (mean 0.97 vs 0.89, 3.6 vs 2.93, 0.26 vs 0.17 mmol/L, respectively), but the differences were not statistically significant (Figure 1G**,** all *P*>0.05).

**Morphologic studies of liver and adipose tissue of *TM4* KO mice**

Liver tissue was observed under optical microscopy after HE staining. In wild-type mice, the hepatic lobule structure was clear and complete. The hepatic sinusoids were clear, and hepatocytes were arranged in an orderly manner from the central vein to the periphery in a radial manner. Hepatocytes contained abundant cytoplasm, with the round nucleus located in the centre (Figure S3A).

In *TM4* knockout mouse liver tissue, the integrity of the lobular structure was maintained, but hepatic sinusoid stenosis and hepatocyte swelling could be observed. The hepatocyte cytoplasm was filled with large round fat vacuoles, with the nucleus pushed aside, and the cell size was significantly larger than that of the WT control. Moreover, a few necrotic hepatocytes were observed, along with inflammatory cell infiltration (Figure S3B).

A transmission electron microscope was used to verify that the structure of hepatocytes in WT mice was normal. The nucleolus, nuclear membrane, mitochondria, Golgi apparatus, and endoplasmic reticulum had normal structures (Figure S3C). In contrast, *TM4* KO mice had abundant lipid deposition in their hepatocyte cytoplasm, with mitochondrial swelling and mitochondrial crista deformation and fracture (Figure S3D). However, the Golgi apparatus and endoplasmic reticulum were normal.

The mice were fed a high-fat diet for 24 weeks before they were euthanised. Epididymal fat samples were embedded in paraffin, sliced, and HE stained. Under optical microscopy, the size of *TM4* KO mouse adipocytes was significantly larger than that of the wild-type control (Figure S3E, S3F). Immunohistochemistry with the macrophage-specific marker F4/80 showed brown staining and typical crown-like structures in the intercellular space of adipocytes, suggesting macrophage infiltration (Figure S3H).

Our previous results demonstrated that *TM4* KO mice developed impaired glucose tolerance (Figure 1D) on a high-fat diet. A serum protein chip showed that the serum insulin of *TM4* KO mice was significantly elevated (data not shown), suggesting insulin resistance. AKT is an important protein in the insulin signalling pathway, since AKT phosphorylation decreases in the case of insulin resistance. Therefore, we examined phosphorylated AKT expression in mouse visceral adipose tissue by western blotting and found that AKT phosphorylation was significantly decreased in the visceral adipose tissue of TM KO mice (Figure S4C).

**TM4 counterregulates Nur77, IKKβ, and NF-κB: *TM4* KO mice**

Previous *in vitro* results demonstrated that Nur77 was a TM4 interacting protein. Reduction of TM4 expression could induce the expression of downstream Nur77, IKK-β, and NF-κB. During the course of this research, we tried to verify this in the liver and visceral adipose tissue of *TM4* KO mice through western blot analysis.

Nur77 expression was significantly increased in the liver (Figure S4D) and epididymal fat (Figure S4E) of *TM4* KO mice compared with that of the WT controls. The expression of phosphorylated IKK-β was also significantly increased in the liver (Figure S4F) and epididymal fat (Figure S4G) of *TM4* KO mice compared with that of the WT controls. Moreover, the expression of phosphorylated NF-kB was significantly increased in the liver (Figure S4F) and epididymal fat (Figure S4G) of *TM4* KO mice compared with that of the WT controls.

**The effect of high glucose and high fat on TM4 expression**

In vascular smooth muscle cells, high glucose inhibited the expression of TM4 in a dose-dependent manner (Figure S5A). Various concentrations (5.6 mM to 30 mM) of glucose were added to the culture medium of human umbilical vein endothelial cells (HUVECs). HUVECs were harvested after 24 h, and then western blotting was performed to detect TM4 expression. Under different glucose concentrations, TM4 expression showed no significant difference (Figure S5B), indicating that TM4 is not sensitive to sugar stimulation in HUVECs.

Next, free fatty acids (FFAs) were applied to HUVECs. BSA was used as a negative control. HUVECs were harvested after 24 h, and western blotting was performed to detect TM4 expression. As shown, when the free fatty acid concentration was less than 1.0 mol/L, TM4 protein levels did not change significantly. When the free fatty acid concentration reached 2.0 mol/L, TM4 protein expression was significantly reduced (Figure S5C).

**The effect of PS-341 on** **insulin resistance: hyperinsulinaemic-euglycaemic clamp test**

The right jugular veins of *db/db* mice were catheterized for the hyperinsulinaemic-euglycaemic clamp test. Insulin was infused continuously, and blood glucose was measured every 5 minutes (Figure S6a). Approximately 60 minutes later, the blood glucose of the mice in each group stabilized, and then a 10% glucose solution was infused based on the blood glucose levels to maintain blood glucose homeostasis (Figure S6b). The glucose infusion rate (GIR) at 60~120 minutes was further analysed (Figure 3I). The GIR of the PS-341 gavage group was significantly higher than that of the PBS gavage group (*P*＜0.05), the GIR of the PS-341 iv group was significantly higher than that of the PBS iv group (*P*＜0.05), and the GIR of PS-341 gavage group was significantly higher than that of the PS-341 iv group (*P*＜0.05). The results suggest that PS-341 can improve insulin resistance and that gavage is more effective than intravenous administration.

**TM4 expression in human omental adipose tissue**

The general characteristics of the study participants who provided omental adipose tissue during laparoscopic cholecystectomy are presented in Table S3. TM4 expression in human omental adipose tissue of 12 participants is shown in Fig S7.

**Supplementary Figures**

**
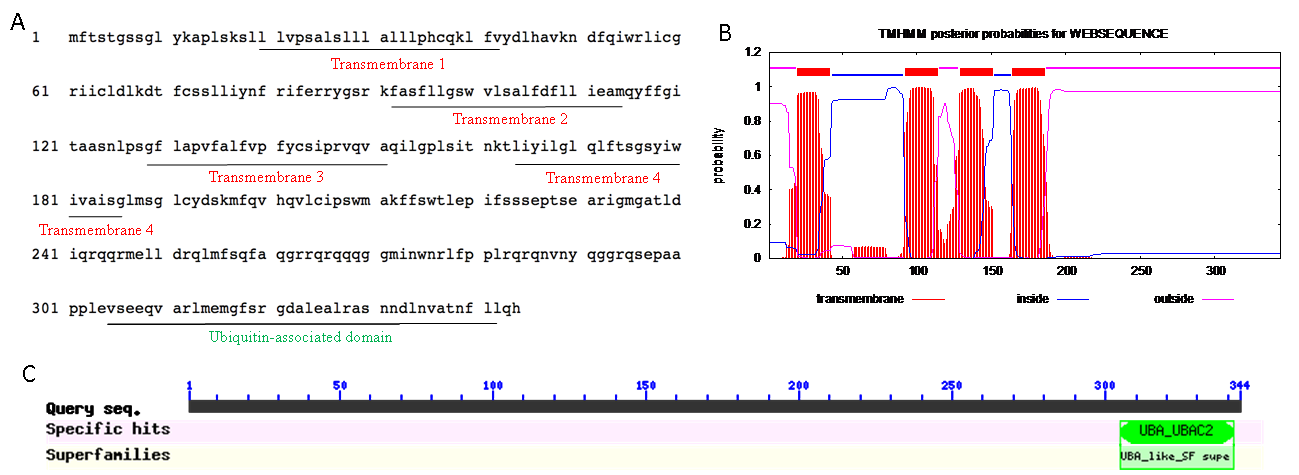
**

**Supplementary Figure 1 Online bioinformatics analysis of the TM4 protein**

**
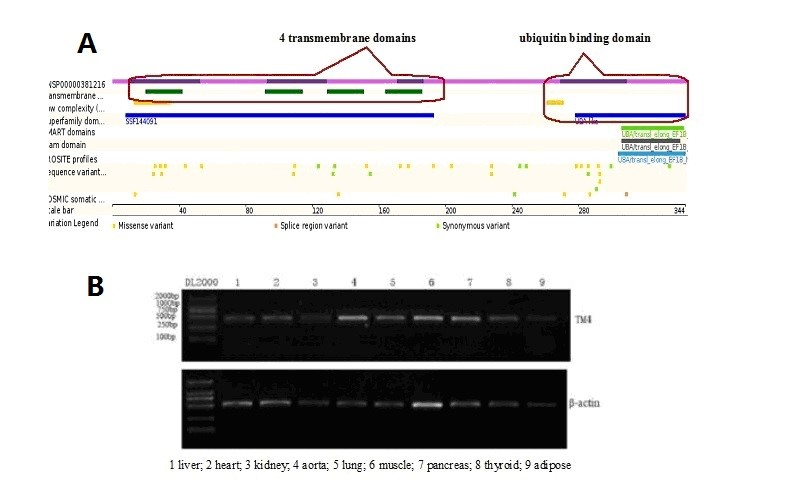
**

**Supplementary Figure 2 The expression profiles of TM4**

**Figure S2A**: Bioinformatics analysis of TM4 protein. **Figure S2B**: The expression profiles of TM4.

**
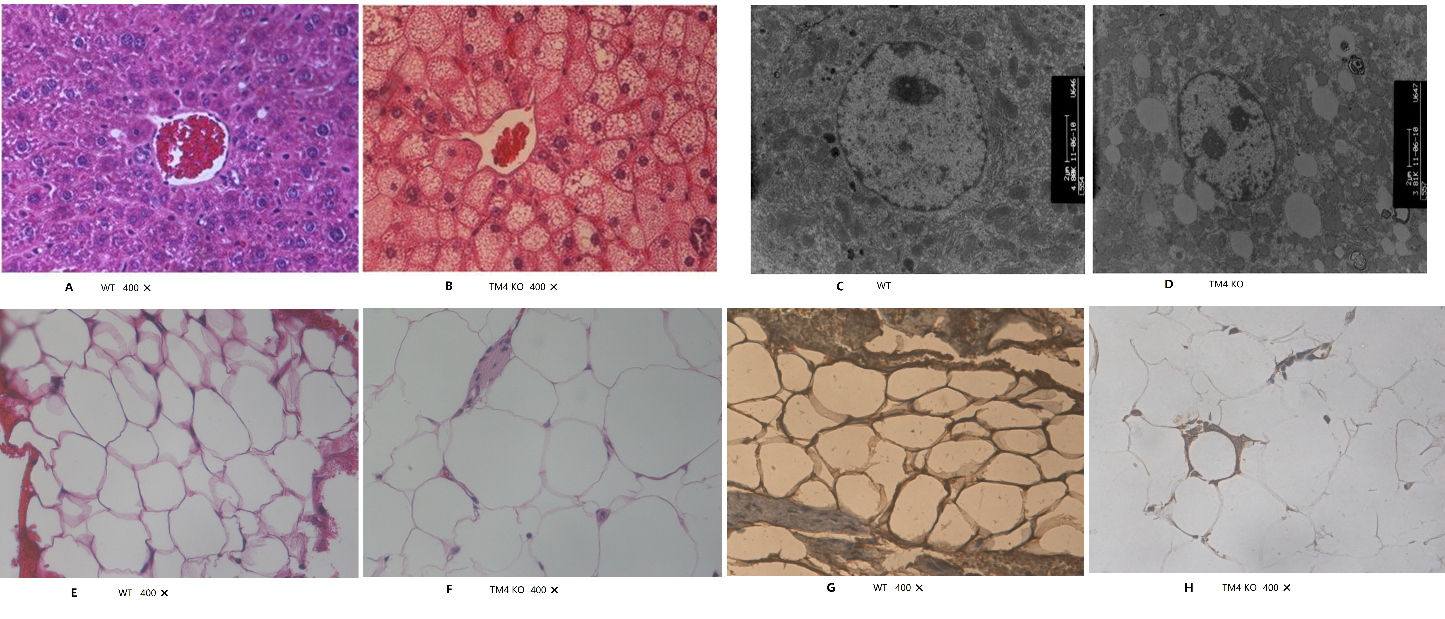
**

**Supplementary Figure 3 Low-grade inflammation in the liver and adipose tissues of *TM4* KO mice**

**Figure S3A, S3B:** HE staining of the mouse liver tissue**.** (A) Hepatocytes of WT mice with a regular distribution under microscopy (HE staining, 400×). (B) Hepatocytes of *TM4*^-/-^ mice demonstrated diffuse fat deposition (HE staining, 400×). **Figure S3C, S3D:** Mouse liver tissue under transmission electron microscopy. **Figure S3E, S3F:** HE staining of mouse visceral fat tissue. (E) WT visceral adipose tissue (HE staining, 400×). (F) The average adipocyte size in *TM4* KO mice was larger than that in WT mice (HE staining, 400×). **Figure S3G, S3H:** Immunohistochemistry staining of F4/80 in mouse visceral fat tissue. (G) The visceral adipose tissue of WT (400×). (H) The visceral adipose tissue of *TM4* KO mice (400×).

**
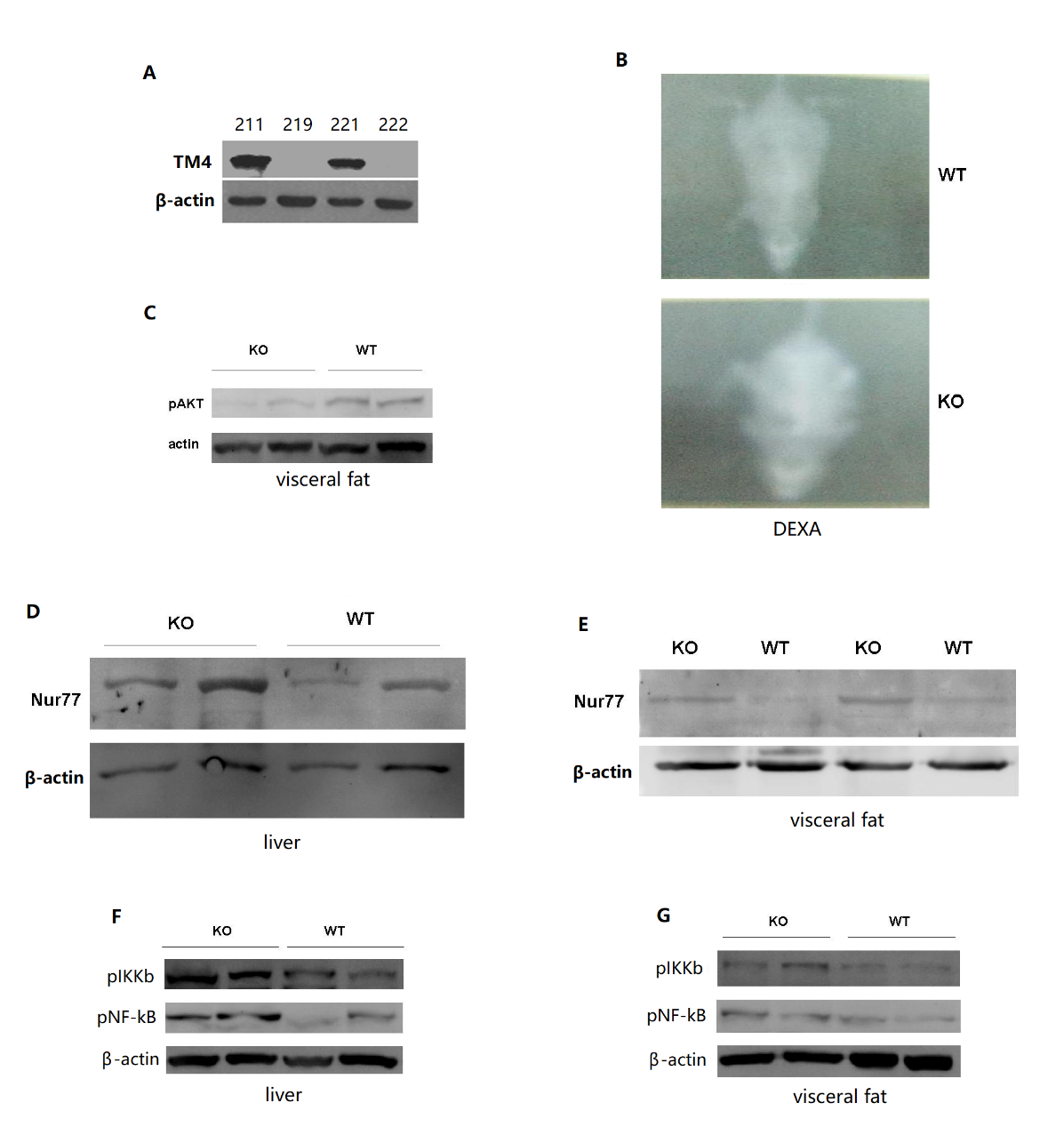
**

**Supplementary Figure 4 Expression of Nur77, IKKβ, NF-κB and pAkt in liver and visceral fat tissues of *TM4* KO mice**

**Figure S4A:** Verification of *TM4* KO. **Figure S4B:** Mouse body composition under DEXA. **Figure S4C**: Phosphorylated AKT expression in the visceral fat of *TM4* KO and WT mice. **Figure S4D:** Nur77 expression in the livers of *TM4* KO and WT mice. **Figure S4E:** Nur77 expression in the visceral fat of *TM4* KO and WT mice. **Figure S4F:** Phosphorylated IKK-β and phosphorylated NF-κB expression in the livers of *TM4* KO and WT mice. **Figure S4G:** Phosphorylated IKK-β and phosphorylated NF-κB expression in the visceral fat of *TM4* KO and WT mice.

**
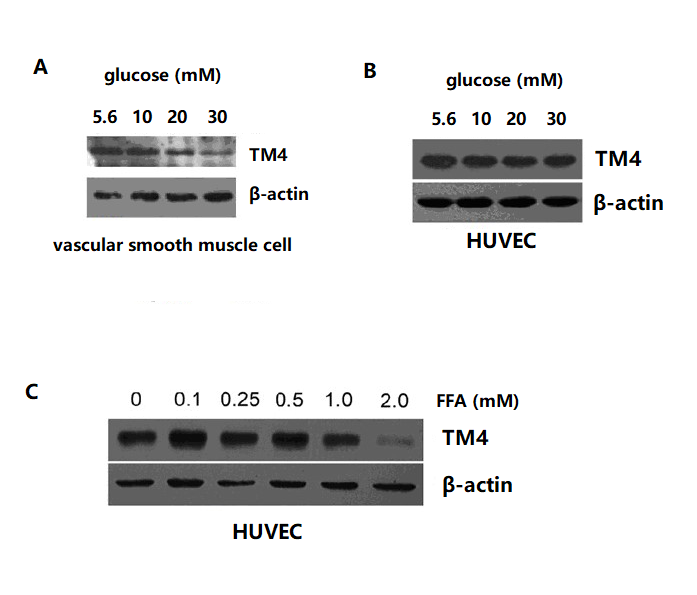
**

**Supplementary Figure 5 The effect of high glucose and FFAs on TM4 expression**

**Supplementary Figure 5A:** High glucose inhibits the expression of TM4 in vascular smooth muscle. **5B:** The effect of high glucose on HUVEC. **5C:** The effect of FFA on HUVEC.

**
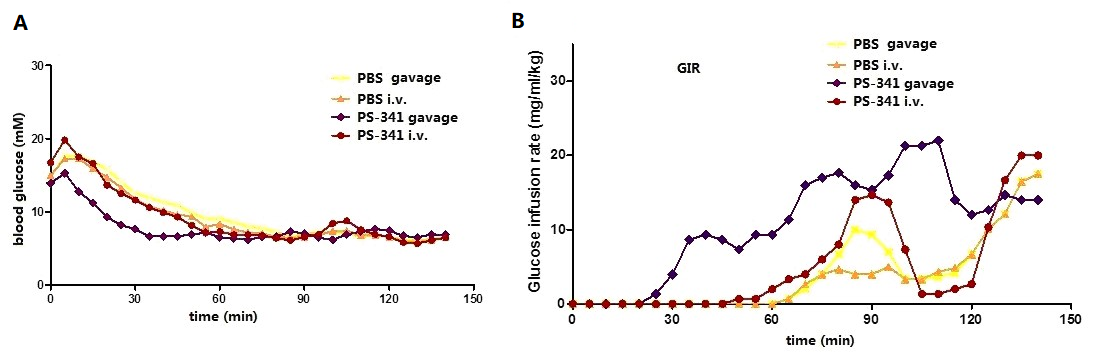
**

**Supplementary Figure 6 Hyperinsulinaemic-euglycaemic clamp test**

**Supplementary Figure 6a:** Blood glucose during the hyperinsulinaemic-euglycaemic clamp test. **6b:** Glucose infusion rate during the hyperinsulinaemic-euglycaemic clamp test.


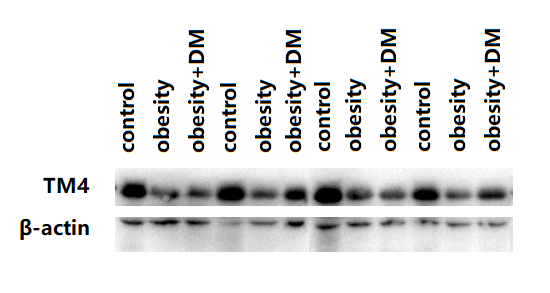


**Supplementary Figure 7 TM4 expression in human omental adipose tissue of 12 participants**

**Table S1** **Hardy-Weinberg tests**

| Group | Major/  minor allele | ALL | | Case | | Control |  |
| --- | --- | --- | --- | --- | --- | --- | --- |
|  |  | Genotype count 11/12/22# | P value | Genotype count 11/12/22# | P value | Genotype count 11/12/22# | P value |
| T2DM | C/T | 2/51/1809 | 0.0111 | 2/50/1722 | 0.0599 | 0/1/87 | 1 |
| NGR | C/T | 3/90/3297 | 0.0043 | 2/19/920 | 0.0071 | 1/71/2377 | 0.4199 |

**Table S2 Half-life of TM4 after PS-341 intervention**

| No. | Time | Relative Protein Content | | |
| --- | --- | --- | --- | --- |
|  |  | HUVEC | CHX | CHX+PS341 |
| 1 | 0 h | 1 | 1 | 1 |
| 2 | 0.5 h | 0.976286 | 0.6858677 | 0.800982411 |
| 3 | 1 h | 0.9742425 | 0.5403126 | 0.753072774 |
| 4 | 2 h | 1.0056677 | 0.3851557 | 0.663700337 |
| 5 | 4 h | 0.9840947 | 0.1603517 | 0.553837796 |
| 6 | 8 h | 0.9444889 | 0.0844368 | 0.45517498 |
| 7 | 16 h | 0.915453 | 0.0581673 | 0.392196897 |

**Table S3 General characteristics of the study participants who provided omental adipose tissue during laparoscopic cholecystectomy**

| Group | Gender | Age(yr) | Metabolic complications | Height  (cm) | Weight  (kg) | BMI  (kg/m2) | SBP/DBP  (mmHg) | FPG  (mmol/l) | 2 hPG  (mmol/l) | HbA1c  (%) |
| --- | --- | --- | --- | --- | --- | --- | --- | --- | --- | --- |
| 1 | female | 35 | / | 164 | 55 | 20.45 | 92/60 | 4.5 | UA | 5.0 |
| 1 | female | 46 | / | 168 | 57 | 20.20 | 113/72 | 5.0 | UA | 5.3 |
| 1 | male | 52 | / | 170 | 65 | 22.49 | 98/70 | 4.3 | UA | UA |
| 1 | female | 57 | / | 160 | 56 | 21.88 | UA | UA | UA | UA |
| 2 | female | 21 | obesity | 161 | 101 | 38.96 | 120/75 | 5.0 | 4.3 | 5.7 |
| 2 | female | 36 | obesity | 168 | 88 | 31.18 | 132/82 | 5.7 | 8.9 | 5.3 |
| 2 | female | 25 | obesity | 165 | 87 | 31.96 | UA | 5.9 | 6.2 | 5.4 |
| 2 | female | 22 | obesity | 168 | 96 | 34.01 | 117/76 | 4.2 | 4.1 | 5.2 |
| 3 | male | 50 | obesity, diabetes, hypertension | 172 | 130 | 43.94 | 123/91 | 8.4 | 14.6 | 7.2 |
| 3 | female | 30 | obesity, diabetes, hypertension | 168 | 100 | 35.43 | 155/109 | 7.4 | 14.8 | 7.6 |
| 3 | male | 33 | obesity, diabetes | 165 | 97 | 35.63 | 126/80 | 16.0 | 22.4 | 11.0 |
| 3 | female | 41 | obesity, diabetes | 159 | 105 | 41.53 | 132/88 | 12.9 | 20.6 | 10.7 |

Group 1: control group (nonobese participants with normal glucose tolerance, n=4); Group 2: obesity group (obesity with normal glucose tolerance, n=4); Group 3: obesity + DM group (obesity with type 2 diabetes, n=4). BMI: body mass index; SBP: systolic blood pressure; DBP: diastolic blood pressure; FPG: fasting plasma glucose; 2 h PG: 2-hour postprandial plasma glucose; UA: unknown.
